# The Relevance of Spinal Muscular Atrophy Biomarkers in the Treatment Era

**DOI:** 10.3390/biomedicines12112486

**Published:** 2024-10-30

**Authors:** Marianna Maretina, Valeria Koroleva, Lyudmila Shchugareva, Andrey Glotov, Anton Kiselev

**Affiliations:** 1 D.O. Ott Research Institute of Obstetrics, Gynecology and Reproductology, 199034 Saint-Petersburg, Russia; marianna0204@gmail.com (M.M.); anglotov@mail.ru (A.G.); 2 Municipal Hospital for Children No. 1, 198205 Saint-Petersburg, Russia; pvd21@bk.ru (V.K.); neurodoctor@mail.ru (L.S.); 3 Department of Pediatric Neuropathology and Neurosurgery, North-Western State Medical University Named After I.I. Mechnikov, 191015 Saint-Petersburg, Russia

**Keywords:** spinal muscular atrophy, *SMN1* gene, *SMN2* gene, biomarkers, prognostic criteria, effectiveness of pharmacotherapy

## Abstract

Spinal muscular atrophy (SMA) is a severe neuromuscular disorder that currently has an approved treatment for all forms of the disease. Previously, biomarkers were primarily used for diagnostic purposes, such as detecting the presence of the disease or determining a specific clinical type of SMA. Currently, with the availability of therapy, biomarkers have become more valuable due to their potential for prognostic, predictive, and pharmacodynamic applications. This review describes the most promising physiological, functional, imaging and molecular biomarkers for SMA, derived from different patients’ tissues. The review summarizes information about classical biomarkers that are already used in clinical practice as well as fresh findings on promising biomarkers that have been recently disclosed. It highlights the usefulness, limitations, and strengths of each potential biomarker, indicating the purposes for which each is best suited and when combining them may be most beneficial.

## 1. Introduction

Proximal spinal muscular atrophy 5q (SMA) is a severe autosomal recessive neuromuscular disease characterized by progressive symptoms of muscular weakness and paralysis due to degeneration of α motor neurons in the anterior horns of the spinal cord [1]. The disease is associated with pathogenic mutations in the *SMN1* gene, which results in the loss of function of the survival motor neuron protein. This, in turn, leads to denervation of skeletal muscles and the development of muscle atrophy [2]. SMA occurs with the incidence of 1 per 6000–10,000 live births, with a carrier frequency of about 1 in every 40–60 people [3].

Based on the age of symptoms onset and disease severity, SMA is subdivided into types 0–IV. Type 0 is the most severe form with embryonic onset. Type I is also an acute form (Werdnig–Hoffmann disease) defined by generalized muscle weakness with onset in the first months of life and respiratory distress. Type II is an intermediate form. Type III (Kugelberg–Welander disease) is a milder form established after the age of 18 months and characterized by preserved walking ability. Finally, there is SMA type IV, which includes a minor group of patients with first symptoms appearing in adulthood [4,5]. A more detailed division into subgroups (I a–c, II a–b, III a–b) actually indicates that referring to a certain type is conditional enough. SMA phenotypes represent a continuous spectrum, ranging from the most severe to the mildest forms [6].

Homozygous mutations within the *SMN1* gene underlie the development of all forms of 5q-SMA. The *SMN1* gene is mapped on chromosome 5 at locus 5q13 and has a centromeric copy, the *SMN2* gene [2]. The *SMN1* gene produces a full-length transcript, while the *SMN2* gene, due to a point mutation in exon 7 (c.840 C>T), generates a pre-mRNA that undergoes aberrant splicing [7]. This substitution creates a splicing silencer instead of a splicing enhancer, leading to a decline in full-length (FL) mRNA transcript level and an increase in the SMN mRNA, lacking exon 7 (Δ7-SMN) [8]. This results in a decrease in the production of a full-sized functional protein. While non-SMA individuals may totally lack the *SMN2* gene, in SMA patients *SMN2* is the only source of the SMN protein [9]. The *SMN2* gene copy number was found to inversely correlate with SMA severity and was shown to be an important factor, affecting disease progression and treatment efficacy [10,11,12,13]. For decades, research into SMA treatment methods has focused on the *SMN2* gene as a key therapeutic target. In 2016, *SMN2* splicing modulator oligonucleotide nusinersen (Spinraza^®^, Cambridge, MA, USA) was approved by the FDA and represented the first certified drug to treat SMA [14,15]. Later, onasemnogene abeparvovec (Zolgensma^®^, Durham, NC, USA), representing an AAV9 vector containing the *SMN1* gene, and small-molecule *SMN2* splicing modulator Risdiplam (Evrysdi^®^, San Francisco, CA, USA) were approved for treatment of SMA as well [16,17]. Importantly, valuable factors such as the number of *SMN2* gene copies and the age of the patient at the time of treatment were addressed when establishing the strategies of therapeutic intervention in patients with SMA [18]. Despite the reported efficacy of the above-mentioned therapeutics, a notable proportion of SMA patients who undergo treatment continue to be non-responders [19,20].

**Figure 1:**
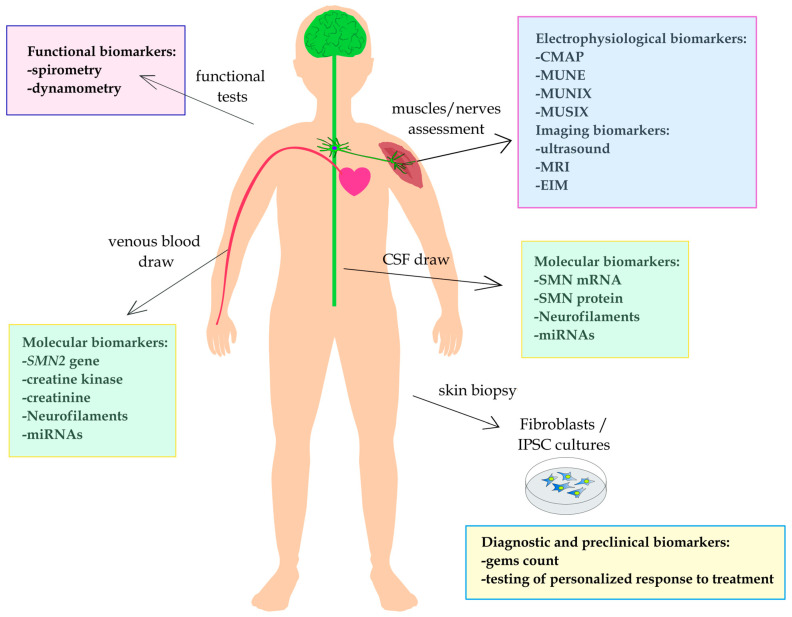
The origin of prospective SMA biomarkers in human organism. Informative biomarker measures can be derived from different tissues. Molecular biomarkers are determined in blood, CSF, and patient-derived cell cultures. Electrophysiological and imaging biomarkers reflect muscle and nerve state. Functional biomarkers are obtained from physiological tests.

In the SMA treatment era, when approved drugs are introduced, and new therapies are developed, biomarkers of the disease can be particularly helpful. They can be used to predict therapeutic responses and monitor the effect of therapy as well as determine if a response exists, even if it is not detected by standard clinical outcome measures. Moreover, molecular modifiers of SMA severity that may influence treatment outcome and thus implement biomarker functions are of particular interest. A single biomarker that may universally fulfill all these roles is desirable but not quite feasible, especially in the context of multiple molecular variants or candidate biomarker proteins. Indeed, putative biomarkers include *SMN2* gene polymorphisms, *SMN* transcripts and protein, transcription regulators (miRNAs), muscle condition indicators (creatine kinase and creatinine), or neuron integrity markers (neurofilaments). Also, SMA biomarkers are regarded among more classical electrophysiological, functional, and imaging measurements [18,21] (Figure 1). Depending on the age or functional status of the SMA patient as well as on the goals of biomarker use, specific biomarker measurements can be determined in certain tissue.

To date, clinical measures of motor function have been implemented in the monitoring of SMA progression and treatment response. Involvement in the practice of new biomarkers, based on data obtained from the exploration of molecular factors in SMA patients’ blood, cerebrospinal fluid (CSF), and muscles, may provide a better understanding of the pathological processes in a particular SMA patient and increase prognostic power for treatment decisions.

## 2. Physiological Biomarkers

### 2.1. Electrophysiological Biomarkers

In recent years, multiple putative molecular biomarkers for SMA have been discovered. At the same time, electrophysiological biomarkers are routinely applied in clinical studies to monitor the functional status of motor units in patients with neuromuscular diseases [22].

Electrophysiological measures, such as the compound muscle action potential (CMAP), motor unit number estimation (MUNE), motor unit number index (MUNIX), and motor unit size index (MUSIX), have been shown to be the most reliable.

#### 2.1.1. Compound Muscle Action Potential

CMAP demonstrates the response of the muscle to stimulation of the motor nerves. CMAP amplitude reflects the summation of all activated motor fibers capable of conducting impulses. In SMA patients, CMAP values have been correlated with age, SMA type, and *SMN2* gene copy number [13,23]. The maximum CMAP amplitude correlated in one study with the Modified Hammersmith Functional Motor Scale (MHFMS) motor function scores and differed significantly between ambulatory and non-ambulatory SMA patients [24]. In another study, the maximum CMAP at the moment of the initial measurement was shown to be most prognostic regarding functional outcome (*p* < 0.0001). It was considered as a predictive factor defining expectations about clinical improvement after therapeutic interventions [13]. A study of symptomatic SMA patients before and after therapy with nusinersen or onasemnogene abeparvovec demonstrated that CMAP amplitudes increased after treatment and can serve as a predictor of motor recovery [25,26,27]. Weng and colleagues investigated the CMAP in SMA patients detected through a newborn screening program and found that a rapid decrease in CMAP amplitude served as an early predictor of symptom onset [28]. Also they observed that patients with a pre-treatment CMAP amplitude ≥2 mV demonstrated a higher increment in post-treatment CMAP than those with a pre-treatment CMAP amplitude <2 mV [28]. Thus, pre-treatment CMAP values and changes in CMAP after treatment can predict treatment outcomes.

The CMAP amplitudes are known to be well tolerated by younger patients, easy to perform, and non-invasive. At the same time, due to compensatory mechanisms that may occur during slow, progressive denervation, when surviving motor nerves enlarge and sprout, changes in the CMAP response may not always be detectable [29].

#### 2.1.2. Motor Unit Number Estimation

In this respect, MUNE provides a more direct assessment of the number of functional motor units. MUNE is calculated as the CMAP amplitude divided by the mean single-motor-unit action potential (SMUP) amplitude, which represents the number of muscle fibers innervated by a single motor neuron. This number can be counted using graded stimuli that activate individual units in an “all-or-nothing” response fashion [30]. Equally to CMAP, MUNE was found to be reduced in SMA type I and II patients, correlated with age, SMA type and SMN2 gene copy number. It was also shown to increase in patients after therapeutic intervention [13,26]. At the same time, MUNE scores demonstrated a decline in SMA type III children even after treatment with nusinersen. This indicates that the age and severity of denervation at the time of treatment initiation may considerably determine the response to therapy [31,32]. The authors argued that this observation is expected, as distal peripheral reinnervation can be more challenging to rescue in elder patients with a larger body mass and the need for appropriate SMN levels at distal terminals [31]. In a number of studies, MUNE has demonstrated higher sensitivity compared to CMAP in detecting and tracking denervating diseases [33]. MUNE techniques are generally known to be non-invasive and well tolerated. However, they are technically challenging and time-consuming and may not be the optimal option for use with younger patients [29].

#### 2.1.3. Motor Unit Number Index and Motor Unit Size Index

Compared to MUNE, MUNIX is more efficient, easier to execute, and less invasive, making it a more suitable technique for use in pediatric populations [34]. MUNIX is estimated based on CMAP and surface EMG measurements. The value of MUNIX is proportional to the number of motor units rather than providing information about the actual number of motor units within a muscle, as MUNE does [35]. In SMA patients, MUNIX correlates with muscle strength and disease severity [34,36,37,38]. This technique was shown to be quite sensitive for detecting the early loss of lower motor neurons in SMA patients and monitoring disease progression [34].

MUSIX is another electrophysiological biomarker that provides information about the size of certain motor units in relative rather than absolute value. It is calculated as the CMAP amplitude divided by the MUNIX for a specific muscle [35]. MUSIX values were shown to be increased in SMA patients compared to healthy controls, indicating reinnervation due to compensatory nerve sprouting [34,37]. MUNIX and MUSIX were shown to be more sensitive indicators of compensatory axonal sprouting and the number of functional motor units compared to CMAP [34]. Both techniques have also been used as outcome measures to determine functional changes in the hand muscles of SMA patients [32].

Considering the specific characteristics of the aforementioned electrophysiological biomarkers, they can be used in combination with each other. Each one highlights certain aspects of disease pathogenesis and progression.

### 2.2. Functional Biomarkers

#### 2.2.1. Spirometric Measurements

Spirometry is a non-invasive, sensitive, and easy-to-perform test. It can be applied for detecting early changes in pulmonary function as well as the disease progression and for monitoring the effects of medications [39]. The involvement of the respiratory system is a characteristic feature of many neurodegenerative and muscle-wasting diseases, such as SMA, ALS, and DMD [40,41,42]. Respiratory failure is one of the key features of severe forms of SMA and the most important cause of mortality in untreated SMA patients [5]. A previous longitudinal study of DMD and ALS patients showed that changes in spirometric measurements over time can be used as a prognostic biomarker to predict survival time and functional ability. These findings should be considered when planning treatment [41,43]. In SMA patients, poor respiratory function at birth has been associated with reduced survival [44].

Important spirometric measures for neuromuscular diseases include vital capacity (VC), which is defined as the maximum amount of air exhaled when blowing out at a steady rate. Forced vital capacity (FVC) is a similar characteristic but is measured when breathing out as fast as possible. Forced expiratory volume in 1 s (FEV1) is an important measure as well [39,40]. All these indicators of lung function were found to differ significantly between SMA types as well show annual changes in the values of these spirometric measures [5]. The maximum VC in SMA patients was also correlated with prognosis for autonomous breathing [45]. Treatment with nusinersen improved the FVC in SMA children [46,47,48]. The response of respiratory function to the drug was shown to depend on different factors, with the SMA subtype being crucial [47,49]. It was observed that the earlier and longer the treatment was applied, the better the response in respiratory function was [48]. While respiratory function has improved under nusinersen treatment, it has not improved to the same extent as motor function, and therefore, it is worth monitoring closely [50].

The limitations of FVC and FEV1 include the fact that they are voluntary tests that require cooperation from the patient. This makes these techniques unsuitable for the youngest individuals with SMA [51].

#### 2.2.2. Dynamometric Measurements

Grip strength measured by dynamometry is a well-examined indicator of muscle status [52]. Its clinical and prognostic significance has been demonstrated for various pathological conditions, correlating with patient survival time and functional status [52]. In the SMA condition, manual muscle testing using a hand grip dynamometer has demonstrated high reliability for repeated strength measurements. This technique has also shown high sensitivity in evaluating differences in muscle strength between patients with different forms of SMA and within the same patient before and after treatment [53,54,55]. One study found a significant association between motor function and muscle strength as measured by dynamometry. These measurements differs significantly between ambulatory and non-ambulatory patients with SMA [54]. Dynamometry has been considered an important measure of upper-limb function for determining the natural history of SMA and assessing changes in clinical trials [53,56,57].

The convenience of hand-grip dynamometry lies in its non-invasive nature and ease of use, even for patients who are wheelchair-bound. The ability of this technique to obtain quantitative data enables detection of even small changes in muscle strength [58]. At the same time, dynamometry may be difficult to perform on the youngest patients, as this method requires cooperation. It may also be problematic to use on those who are extremely weak and lack the strength to overcome the resistance of a dynamometer [59,60].

## 3. Imaging Biomarkers

### 3.1. Ultrasound Measurements

Another technique that has been shown to correlate with muscle strength and values obtained through dynamometry in patients with SMA is muscle ultrasonography [60]. This method has been shown to be well tolerated by patients, requiring minimal cooperation and allowing for examination of multiple muscles in a timely fashion [60]. Being non-invasive, safe, and relatively cheap, ultrasound imaging has long proven its relevancy for application to the investigation of neurodegenerative and muscular diseases [61,62]. Increased echo intensity was observed as a characteristic of weakened muscles in patients with reduced muscle thickness due to neuromuscular disorders [61,63]. The sensitivity of ultrasonography may be elevated by applying quantitative assessment of echo intensity through computer analysis of grayscale. This approach is a perspective for follow-up studies on disease progression and therapy [62,64]. Quantitative ultrasound data have shown promising results as a biomarker for Duchenne muscular dystrophy (DMD), amyotrophic lateral sclerosis (ALS), and Charcot–Marie–Tooth disease, demonstrating a correlation with the severity of the diseases [65,66,67,68]. It is important to note that a longitudinal study of DMD patients demonstrated the sensitivity of quantitative ultrasound for detecting progressive changes in muscle structure. Also, an association was observed between muscle echo intensity and muscle strength, ambulation status, and motor function [69]. Quantitative muscle ultrasound was observed to be more accurate than functional tests, such as the 6 min walk test, in detecting early muscle impairments [66].

The feasibility of muscle ultrasound in assessing the severity of muscle damage in SMA type II and III patients was demonstrated by examining the biceps brachii, wrist extensors, quadriceps, and tibialis anterior muscles [60]. The ratio of muscle echogenicity to subcutaneous fat, derived from quantitative ultrasound values, increased with the increasing severity of SMA and was found to be related to muscle strength. This suggests that these quantitative ultrasound measures may serve as a potential biomarker for use in clinical trials for SMA [60]. A longitudinal study on SMA type I patients revealed a decrease in muscle thickness and an increase in echo intensity over time [70]. In adult SMA patients, reduced muscle thickness, elevated muscle echogenicity, and subcutaneous fat thickness were observed compared to controls, with non-ambulatory patients having the most critical values [71].

Ultrasound imaging was reported to be a convenient method for examining diaphragm involvement as a common feature of neuromuscular diseases [42,72]. Ultrasonography enables to reveal the thickness of the diaphragm and other respiratory muscles, as well as the diaphragm motion [72]. Diaphragm ultrasound imaging abnormalities, such as dysmotility patterns of contraction, were defined by a reduced thickening ratio and detected in both SMA type I and adult patients [42,73,74]. These measures provided additional information to the functional respiratory tests. Treatment with nusinersen resulted in improvement of diaphragm ultrasound-determined parameters, which represent a valuable complementary outcome measure [74].

According to multiple studies that implement muscle ultrasonography, this investigation technique enables to distinguish neuromuscular disorder from non-neuromuscular with high sensitivity and specificity. Moreover, it allows for a more precise identification of the type of the disease based on selective vulnerability of affected muscles [75,76]. In this manner, based on muscle echograms, selective muscle involvement was detected, including a dissociation between the soleus and gastrocnemius. It corresponded to magnetic resonance imaging (MRI) data and provided the patient with an early diagnosis of SMA type III and timely treatment [77]. Taking into account the capacity of muscle ultrasonography, which enables to disclose muscle architecture and pathological muscle changes, it may fulfill the role of more challenging methods for muscle tissue assessment, such as MRI or biopsy [78].

Ultrasonography can also measure muscle fasciculation, which was shown to be an important characteristic of diseases caused by the degeneration of anterior horn neurons, including SMA [63,71]. Muscle ultrasound detected fasciculations much more sensitively than EMG [79].

With the help of high-resolution instruments, nerve ultrasonography was also realized in practice for neuromuscular diseases studies [80]. Decline in echogenicity was shown to be a hallmark diagnostic feature of damaged neurons [81]. A study conducted in SMA patients using ultra-high-frequency nerve ultrasound imaging showed a decrease in the number and density of nerve fascicle in SMA type I compared to type II and type III SMA patients and controls [82]. This may reflect the most prominent motor neuron failure in severely affected patients. Changes in nerve size, fascicle number, and fascicle density may be of particular interest to the youngest SMA patients and may be associated with the severity of the clinical course of the disease [82]. Taking into account the very small sample size in this study, further investigations are needed to draw definitive conclusions.

In spite of the multiple advantages of ultrasound, this method has drawbacks, such as differences in echogenicity when using different devices with varying ultrasonic properties. The application of device-specific reference values and the calibration of the machines may ameliorate this situation.

### 3.2. Magnetic Resonance Imaging

In SMA, as well as related neuromuscular diseases, muscle atrophy is accompanied by intramuscular fat accumulation that can be quantitatively detected by MRI [83,84]. The MRI muscle fat fraction measures were negatively correlated with muscle strength in neurogenic and myopathic conditions and were even more sensitive than clinical or myometric measurements [84].

In 1992, Liu et al. demonstrated the utility of MRI of the lower limb as a valuable supplementary diagnostic tool for monitoring patients with SMA [85]. More severe MRI-derived muscle imaging was observed in patients with SMA types I and II, who had significantly increased levels of subcutaneous fat, while patients with SMA type III demonstrated milder signs of fat infiltration [86,87]. MRI-determined muscle volume was correlated with electrophysiological (CMAP and MUNE) and clinical measures (Hammersmith Functional Motor Scale Expanded (HFMSE) and dynamometry) in SMA patients [88,89]. In an MRI-based study of 25 patients with SMA type IIIb, the selective involvement of muscles was analyzed. A correlation was found between the duration of the disease and the severity of involvement in the gluteus maximus and triceps brachii muscles [90].

A negative correlation was observed between the muscle MRI score, assessing fat replacement as well as atrophy, and the HFMSE score in SMA types II and III patients [91,92]. Baseline MRI measurements were associated with changes in the degree of HFMSE value after 15 months of nusinersen treatment [93]. These findings indicate that pre-treatment muscle MRI scores may contribute to predicting motor function changes in response to therapeutic intervention. At the same time, longitudinal muscle imaging data from SMA types II and III patients aged 15.7–52.8 showed that MRI-detected thigh muscles parameters demonstrated disease progression despite stable strength and motor functions [94]. This suggests that quantitative MRI can detect disease progression more sensitively than clinical assessments.

MRI also enables to test peripheral nerve affection that is hardly detectable by electrophysiological methods [95]. Severe generalized nerve atrophy was observed in all patients with SMA types II, IIIa, and IIIb, while fatty muscle degeneration was most pronounced in type II and IIIa SMA [96]. Specific MRI signal patterns have been shown to be unique to particular neurological disorders. MRI measures have been proposed as a feasible biomarker for early microstructural changes in nervous tissue, which could be used to monitor disease progression or treatment. However, further research is needed to confirm these findings [96].

A short protocol for muscle MRI was described, enabling the performance of this method in children without the need for sedation [97]. In general, MRI is a well-tolerated, reproducible technique with good test–retest reliability [88]. Multiplanar imaging of the target tissue as well as its quantitative description and the non-invasive nature of the procedure are undeniable advantages of MRI. However, its high cost is a significant drawback.

### 3.3. Electrical Impedance Myography

Electrical impedance myography (EIM) is a relatively novel, non-invasive method that detects the voltage response to high-frequency, low-intensity electrical currents applied to a targeted muscle [98]. This method enables quantitative assessment of muscle state in neurogenic and myopathic disorders such as ALS, SMA, and DMD. EIM has been considered as a perspective biomarker for monitoring muscle diseases progression or assessing treatment efficacy [99,100,101]. EIM measurements of the biceps brachii and tibialis anterior muscles in 21 SMA patients and 18 healthy individuals allowed clear differentiation between SMA patients and healthy children. Moreover, SMA types II were distinguished from SMA type III patients with high accuracy [102]. A correlation was observed between EIM values and strength measurements assessed by dynamometry [102]. Further analysis demonstrated major differences in muscle maturation in 28 SMA children compared to healthy subjects of the same age in a longitudinal study lasting three years [103]. Progressive positive changes were revealed in EIM parameters for control individuals, while EIM scores for SMA subjects remained static over time, reflecting no gain or significant decline in muscle growth [103]. At the same time, in a prospective study of 26 SMA infants vs. 27 healthy controls, correlation between EIM measurements and motor function was observed only in healthy children, with no correlation for patients with SMA. Only one EIM measure out of several tested was able to distinguish between SMA and healthy children in this study [104].

More encouraging results were observed in a mild SMA mouse model. The correlation between EIM values and CMAP, SMN levels, and motor neuron counts was demonstrated in the spinal cord of both SMA and wild-type mice [105]. In this study, EIM exhibited even more sensitivity than CMAP and MUNE assessments. Further investigations in a severe SMA mouse model showed EIM measurements’ sensitivity to treatment effects [106]. EIM values were correlated with CMAP and MUNE measures and were increased in oligonucleotide-treated SMA mice compared to sham-treated mice [106].

The painlessness of EIM along with its need for minimal patient cooperation and its applicability to any superficial muscle and rapidity of application make this technique attractive for use in SMA muscle outcome and dynamic measurements. Although the results of some reported studies are taken into account, further research on SMA patients may be required to better understand the sensitivity of EIM.

## 4. Molecular Biomarkers

### 4.1. SMN2 Gene Copy Number

The capacity of the *SMN2* gene to produce a certain amount of full-length *SMN* mRNA and protein makes it the principal molecular modifier of SMA severity. The complete absence of *SMN2* gene copies in an *SMN1*-null background is embryonically lethal [107]. The quantity of *SMN2* copies in SMA patients typically ranges from one to four, and in rare cases, it can be as high as eight [108]. An increased *SMN2* gene copy number may be generated by duplication or conversion of the *SMN1* gene into *SMN2* and alleviates the severity of the disease [109]. An inverse correlation was observed between the number of *SMN2* gene copies and the severity of SMA. One *SMN2* copy is typical for the most severe type 0 SMA, two copies are generally characteristic for type I SMA, three copies are typically found in most patients with type II SMA and also in a significant proportion of type III patients, while four *SMN2* copies are most commonly detected in type III and type IV SMA patients [108,110,111]. A large amount of data on this correlation has enabled the calculation of the likelihood rate to predict the development of a certain SMA type, based on *SMN2* quantification in conjunction with the clinical picture.

An elevated number of *SMN2* gene copies was found in asymptomatic carriers of homozygous deletion in the *SMN1* gene [9,112,113,114]. Amelioration of the SMA phenotype by increasing *SMN2* copies was confirmed in SMA model mice carrying various copy number of the human *SMN2* transgenes on a Smn-knockout background [115]. One or two *SMN2* gene copies were associated with a severe SMA phenotype in mice, a diminished amount of motor neurons, and a lethal outcome at 6–8 days of life, while the presence of eight copies of *SMN2* transgene completely rescued the SMA phenotype [115,116].

Also, a correlation was observed between *SMN2* gene copy number and the duration of SMA patients’ survival as well as their age at onset [110,117,118,119]. The amount of *SMN2* gene copies correlated with MUNE, CMAP, and functional status [13,120]. Moreover, the *SMN2* copy number was associated with the need for scoliosis surgery, respiratory failure, and loss or preservation of walking ability in SMA type III [111,119]. A dependence between the *SMN2* gene copy number and the HFMSE scores has also been revealed [120,121].

Importantly, studies on the pharmacological treatment of SMA patients revealed that a higher amount of *SMN2* copies enhanced the effect of the investigated therapeutic agents [122,123,124]. Thus, the *SMN2* gene copy number was considered a possible predictor of the treatment effect in SMA patients for a long time. Nowadays, with existing pathogenetic therapy, there are more data for *SMN2* contribution to therapeutic outcome. The functional outcomes in SMA patients treated with nusinersen prior to symptoms onset and possessing three copies of the *SMN2* gene were generally superior to those in participants with two *SMN2* copies [125]. The changes in the Children’s Hospital of Philadelphia Infant Test of Neuromuscular Disorders (CHOP INTEND) and HINE-2 values from baseline to 12 months of nusinersen treatment were highly dependent on the number of copies of *SMN2* gene [126]. Therefore, the number of *SMN2* gene copies, together with the age at baseline and the severity of the condition, contributed to predicting the change in CHOP INTEND and HINE-2 scores after 12 months of treatment [126]. Faster gain of functional endpoints was detected in SMA children who had three *SMN2* copies compared to those who had two copies after presymptomatic intervention of onasemnogene abeparvovec [127,128]. In a group of SMA children who were treated with nusinersen before the age of three years, patients with two *SMN2* gene copies demonstrated worse motor, respiratory, and orthopedic characteristics than those with three *SMN2* gene copies after 36 months of therapy [11].

In spite of a great amount of data reflecting the *SMN2* gene copy number impact on SMA phenotype, progression, and therapeutic outcomes, there are numerous discrepant cases that provide evidences of more complicated pathways involved in regulation of SMA pathogenesis. In profound studies dedicated to the investigation of either three or four *SMN2* gene copies in the context of clinical characteristics in SMA patients, both analyzed groups demonstrated a wide range of phenotypes. Thus, disease severity from SMA type II to asymptomatic adults was observed in patients with four *SMN2* copies, and from SMA type I to SMA type IV, phenotypes were reported for those patients who carried three *SMN2* copies [12,114]. Cases of families with marked differences in the SMA phenotype in siblings with the same number of *SMN2* gene copies have been reported as well [9,113,129]. Less than five *SMN2* copies may be revealed in asymptomatic carriers of a homozygous deletion of the *SMN1* gene, while six *SMN2* copies do not always rescue from SMA symptoms [109,130,131]. Such phenotype–genotype discrepancies may be determined by differences in expression between *SMN2* copies, caused by point mutations, aberrant methylation, or the influence of SMN-interacting modifiers [132].

*SMN2* gene copies are known to be not identical due to subtle mutations that can modify the SMA phenotype [133,134]. A c.859G>C variant was described in the *SMN2* gene of SMA patients who developed milder symptoms than expected with their *SMN2* copy amount [135,136,137]. This substitution was found to enhance inclusion of exon 7 into *SMN2* transcripts [138]. Variants A-44G, A-549G, and C-1897T in intron 6 of the *SMN2* gene were found to be associated with milder SMA [133,139]. Actually, being characteristic for the *SMN1* gene, they were found in the *SMN2* gene. This can be explained by gene conversion events [140]. Recently, a mild case of SMA was described with a single *SMN2* gene copy and a positive splice mutation c.628-3T>G in intron 4 [141].

Decreased methylation levels of CpG islands located in the *SMN2* promoter region were also shown to be correlated with milder SMA [142]. An association of methylation at several *SMN2* CpG sites with SMA severity as well as with *SMN2* full-length transcript level was described [143].

These observations suggest that, in addition to the number of *SMN2* copies, other factors should probably be taken into account when evaluating the prognostic and predictive value of *SMN2*. Nevertheless, the importance of considering the number of copies of the *SMN2* gene remains undeniable.

### 4.2. SMN Transcripts Level

At first glance, the *SMN* transcript level seems to be a more sensitive biomarker than the *SMN2* gene copy number, as it results from the expression of *SMN2* gene copies with respect to all cDNA variants, influence of methylation, and other factors. However, considering the tissue-specific nature of *SMN* expression and the various variations in *SMN* transcript analysis, the use of *SMN* mRNA as a prognostic or response biomarker presents a significant challenge. At present, there is no universally accepted method for measuring *SMN* mRNA, nor is there a universal biomarker based on *SMN* mRNA. Sumner and colleagues used quantitative reverse transcriptase PCR (qRT-PCR) to detect the levels of full-length exon 7-containing SMN transcripts (FL-SMN) and SMN transcripts lacking exon 7 (∆7) in the peripheral blood of SMA patients, carriers, and healthy individuals. The levels of FL-SMN mRNA were correlated with the numbers of *SMN1* and *SMN2* genes, while the levels of ∆7-SMN mRNA were strongly dependent on the *SMN2* copy number [144]. Another study found significant differences between groups of patients with type I SMA and controls and between patients with types I–III SMA and control groups in terms of the levels of FL-SMN and Δ7-SMN mRNAs [145]. Real-time PCR was used to detect these molecules, and the results were normalized based on the total RNA concentration added to the reverse transcription reaction. None of these biomarkers enabled researchers to distinguish differences in mRNA levels between SMA type I, II, and III patients [145]. The ratio of FL-SMN to Δ7-SMN was proposed as a valuable parameter, distinguishing between SMA patients and carriers as well as between SMA patients and controls [113,146]. FL-SMN and Δ7-SMN transcripts were determined by means of real-time PCR with a reference gene; no correlation between FL-SMN transcripts level and *SMN2* copy number was observed in SMA patients’ blood samples [146]. Quantitative fluorescence (QF) analysis of the FL-SMN and of the Δ7-SMN mRNA normalized to two reference transcripts revealed an increase in the levels of FL, Δ7, and total *SMN2* transcripts in a linear progression with an increase in *SMN2* gene copy number [147]. Furthermore, FL, Δ7, and total *SMN2* mRNA levels but not the FL/Δ7 ratio were inversely correlated with SMA severity [147]. The level of FL-SMN transcripts detected by absolute real-time PCR was significantly lower in the leukocytes of SMA patients compared to controls and differed according to the severity of SMA symptoms [124,148]. Further study found that the level of FL-SMN transcripts, analyzed by same method, increased significantly in the blood of salbutamol-treated patients with SMA [126]. The level of FL-SMN2 and Δ7-SMN transcripts in SMA patients showed an association with MHFMS values [148]. Kolb and colleagues used droplet digital PCR (ddPCR) and showed that *SMN* mRNA levels normalized to reference gene expression were lower in SMA infants compared to controls [149]. In our previous study, the mean percentage of FL-SMN mRNA relative to the total sum of FL-SMN and Δ7-SMN mRNA detected by means of semiquantitative and QF RT-PCR was significantly different between blood samples from SMA patients, carriers, and controls [150,151]. This putative biomarker was observed to be more sensitive and reliable than the FL/Δ7 ratio determined by real-time PCR. It demonstrated a response to therapeutic oligonucleotide delivery in SMA fibroblasts correlating with an increase in gems number [150,151]. It is important to note that the percentage of FL-SMN and Δ7-SMN mRNA differs between blood and fibroblasts cells derived from individuals with SMA. The proportion of FL-SMN transcripts is significantly elevated in fibroblast cell lines [150].

In general, the choice of analytical methods, normalization variants, reference genes, and other parameters can significantly affect the results of the analysis. Therefore, it is essential to select them carefully in order to obtain more accurate results. If the analysis is designed with suitable conditions, levels of FL-SMN and Δ7-SMN mRNAs may serve as informative biomarkers that can contribute to determining SMA progression or monitoring treatment response in human samples, cell/animal models, etc. However, SMN mRNA is primarily accessible for investigation in blood samples, while the spinal cord is the main target of SMA therapy. Therefore, the SMN transcript level may be a more appropriate biomarker for pre-clinical use or in CSF when administering nusinersen.

### 4.3. SMN Protein

The level of SMN protein is the primary target for therapeutic interventions. Therefore, measuring SMN protein seems more biologically significant [144]. The SMN protein is found in human cells, diffusely distributed in the cytoplasm and also accumulated in special nuclear structures called “gems” [152,153]. Gems can be clearly seen under a microscope. Both the total level of SMN protein, isolated by molecular methods, and the number of gems, counted on cytogenetic samples, are considered as SMA biomarkers.

The ELISA method is widely used for SMN protein analysis in SMA patients’ blood samples. The SMN protein level determined by this method was found to be significantly decreased in peripheral blood mononuclear cells (PBMCs) of SMA patients compared to healthy controls/carriers. However, there was no significant difference between patients with different types of SMA [148,154,155]. No correlation was found between SMN protein levels and *SMN2* gene copy number, *SMN* transcripts level, or motor functions [148]. Salbutamol treatment did not change the level of the SMN protein in SMA patient blood samples, but valproate and phenylbutyrate increased the SMN level in the blood and in skin fibroblast cultures from these patients [126,155,156]. SMN protein levels determined by ELISA showed high intra-individual variability, up to an 8-fold difference, at different time points [154,157].

Other methods for evaluating the SMN protein level were developed. Thus, the Elecsys platform-based assay by Roche Diagnostics enabled one study to reveal a correlation between the SMN protein and the mRNA levels in SMA type I and II but not in type III PBMCs [158]. The electrochemiluminescence-based immunoassay described by Zaworski and colleagues allowed them to detect the SMN protein in a whole-blood volume as little as 5 μL. A difference in SMN protein level was found between SMA patients and controls, and a correlation between the SMN protein level and the *SMN2* gene copy number was described in one study [159]. The SMN protein level determined by means of this method in different time points did not show significant intra-individual variation [159]. Later, the same group demonstrated an association between the level of SMN protein in blood from SMA patients and the severity of denervation, measured by the maximum ulnar CMAP amplitude [160]. Levels of the SMN protein in the blood correlated with those in the spinal cord in the SMA mouse model. However, neither onasemnogene abeparovoc nor nusinersen increased SMN levels in the blood of SMA patients [159,160].

Gems counting in cultured cells derived from SMA patients is quite sensitive to determining differences between individuals with different *SMN1*/*SMN2* backgrounds and capturing therapeutic responses. But this procedure requires a biopsy in the case of investigating skin fibroblasts, and it is more suitable for pre-clinical studies. A reduced number of gems was observed in fibroblasts from severely affected SMA type I patients compared to milder SMA forms, who in turn demonstrated lower gems amounts than controls [161,162]. An inverse correlation was seen between gems number and SMA severity [161,163]. In the study by Cook and colleagues, increased gems amount but not the *SMN2* gene copy number correlated with a mild disease phenotype in SMA patients with a pathogenic c.5C>G missense variant [164].

An increase in gems number was reported in multiple studies investigating the effects of histone deacetylase inhibitors and aminoglycosides in SMA fibroblast cell lines and iPS-SMA-derived neural cells [165,166,167,168,169,170]. Corresponding results were observed with a molecule that enhanced trans-splicing of *SMN2* transcripts in SMA fibroblast cultures [171]. In our recent study, we demonstrated a significant decrease in gems quantity in SMA type II fibroblast cultures compared to the control, while treatment with antisense oligonucleotides induced an increase in gems number. The number of gems in the cell nuclei correlated with the FL-SMN transcripts level [153]. Thus, gems have proven to be quite effective in screening for new therapeutics in vitro and in controlling the induction of *SMN* expression through the beneficial effects of developed drugs.

Among the disadvantages of measuring SMN protein are its relative cost and increased time consumption compared to mRNA analysis methods. Additionally, the level of the SMN protein appears to exhibit greater variability in the same subject over different time periods. Despite this, the SMA level continues to be a primary and direct indicator of the efficacy of SMA therapy, making it a biomarker that merits particular attention.

### 4.4. MicroRNAs

MicroRNAs (miRNAs) are small, non-coding RNA molecules that bind to specific sequences mRNAs of various genes, leading to alterations in gene expression [172]. About 30% of human genes are considered to be regulated by miRNAs [173]. MiRNA-related pathways were shown to play an important role in motor neurons as well as in skeletal muscles, contributing to the pathogenesis of neurodegenerative and muscular diseases like ALS, SMA, and DMD [174,175,176]. Interestingly, total miRNA malfunction was reported to cause an SMA-like phenotype in a Dicer-deficient murine model [177].

Several miRNAs were found to be differentially expressed in the SMA murine and cellular models compared to controls [177,178,179]. MiR-9, miR-206, and miR-132 were dysregulated in the spinal cord, skeletal muscle, and serum of the SMA mice [179]. MiR-9 and miR-132 function in neuronal differentiation and neurite outgrowth, respectively, and are highly conserved among vertebrates [176,177]. MiR-206 is a muscle-specific miRNA involved in the neuromuscular synapses regeneration, and it was shown to play a neuroprotective role in SMA and ALS mouse models [180,181,182,183]. Aberrant expression of miR-9 and miR-132 was found in the brains of patients with Alzheimer’s disease [184]. MiR-9 was found to be most prominently downregulated in the motoneurons of an SMA model mouse. This miRNA was demonstrated to be a regulator of the level of neurofilaments, which are implicated in motor neuron degeneration [177].

MiR-206 was upregulated in the serum of DMD and ALS patients and in the plasma of Alzheimer’s disease patients, and it was regarded as a potential biomarker of these diseases [185,186,187]. In one study, the abundance of miR-206 changed dramatically over time in mdx mice serum (a model of Duchenne muscular dystrophy), reflecting underlying pathophysiological processes in muscles. The level of this miRNA was restored after intravenous injections with therapeutic AONs [188]. In patients with ALS, miR-206 expression was higher at the moderate stage of the disease compared to more severe stages. This expression correlated with better prognosis and slower disease progression [189]. MiR-206 upregulation was considered as a neuroprotective mechanism through the HDAC4-FGFBP1 pathway in response to denervation of the neuromuscular junction. This pathway was proposed to be initiated by muscle cells to increase reinnervation of endplates and thus to have the ability to modulate the progression of neuromuscular diseases [183]. As these mechanisms are similar to those in SMA, miR-206 can be considered a potential biomarker for the severity of disease progression. In fact, miR-206, along with miR-9 and miR-132, showed dynamic changes in expression at different time points in the serum of SMA-I mice, significantly differing from those in control mice. These miRNAs also displayed a remarkable response to systemic AON treatment, resulting in levels reduced to near-normal values [179]. This makes miR-206, miR-9, and miR-132 promising as biomarkers for treatment response. Importantly, changes in the abundance of miR-206, miR-9, and miR-132 were more significant in the serum compared to the spinal cord or muscle tissue of SMA mice. Expression changes were demonstrated at the pre-symptomatic stage for miR-9 and miR-132 and at the mid-symptomatic stage for miR-206 [179]. In SMA patients, the serum levels of miR-9, miR-13,2 and miR-206 were increased relative to healthy controls, reinforcing the potential for these miRNAs, when measured in serum, as non-invasive biomarkers for this SMA [179,190]. A contrasting trend was observed in the expression of miR-9 and miR-132 in the spinal cords of SMA mice and the CSF of SMA patients, where these miRNAs were downregulated [179,191]. Moreover, elevated expression levels of these microRNAs as well as miR-218 and miR-23a were detected in CSF samples from post–nusinersen-treated SMA patients compared to pre-treated ones [191]. MiR-218 is known to be associated with synaptogenesis, and miR-23a is linked to motor neuron survival. MiR-218 was reported to be abundantly produced by motoneurons, and its lack in mutant mice caused severe symptoms of SMA [192]. The expression of miR-23a was reduced in motor neurons derived from iPSCs of SMA patients. Administration of miR-23a through scAAV9 to the SMA mouse model showed a neuroprotective effect of this microRNA, leading to prolonged survival of mice [193]. Interestingly, upregulation of miR-146a was also revealed in CSF samples from post-nusinersen-treated SMA patients [191]. Previously, miR-146a activity was described as significantly upregulated in SMA iPSC-derived astrocytes and in an SMND7 mice-model spinal cord [194]. This indicates that altered miR-146a expression in astrocytes may contribute to SMA pathogenesis. MiR-146a has been considered as a negative regulator of the synaptic function of spinal motor neurons and as an indicator of potential astrocyte involvement in the progression of SMA and the response to nusinersen treatment. This suggests the additional potential use of miRNAs as biomarkers to explore new aspects of the SMA pathology [191].

Whole-miRNome sequencing of muscle biopsy samples and myoblast/myotube cell cultures obtained from SMA patients revealed five overexpressed miRNAs (miR-1, -133a, -133b, -204-5p, and -208b) that were shared in all groups of samples compared to controls [195]. Notably, four of these miRNAs belong to the muscle-specific myomiRNAs, which play a crucial role in regulating myogenesis and muscle degeneration [195]. These results are in line with the previously observed significant decrease in the expression of myomiR-133a, -133b, and -1 and similar trend for miR-206 in the serum of SMA type II and III patients after 6 months of treatment with nusinersen compared to their own baseline levels [196]. These myomiRNA were also investigated as potential biomarkers in the serum of DMD and ALS patients, demonstrating elevated expression levels in non-treated patients compared to healthy controls [185,197]. Dysregulation of miR-206, miR-133a, miR-133b, and miR-1 was observed in SMND7 mice muscles. This was consistent with the altered expression of these myomiRNAs’ target genes, Pax7, Myod1, and Mef2a, which contribute to muscle cell proliferation and differentiation [190]. Remarkably, miR-206 and miR-133a-3p were pinpointed from 2530 miRNAs by NGS of CSF miRNAs in 34 patients with type II and type III SMA before and 6 months after treatment with nusinersen [198]. These miRNAs were found to have prediction capacity for therapy response. Thus, lower baseline levels of miR-206 and miR-133a-3p correlated with a more pronounced response to nusinersen, and the levels of miR-206 were associated with HFMSE values [198]. Earlier, Bonanno and colleagues also demonstrated that a decline in miR-133a expression level in SMA patients’ serum as a result of nusinersen treatment was associated with an improvement in the HFMSE score [196]. This indicates the promising possibilities of measuring the levels of miR-206 and miR-133a-3p to determine the therapeutic effect. Another miRNA whose level was considered a good predictor of SMA patients’ response to therapy and that was able to display treatment efficacy in real time is miR-34. MiRNA-34 was dysregulated in SMA mice and human SMA iPSC-derived motor neurons and was shown to regulate motor-endplate function [199]. The baseline expression level of miR-34b in CSF from SMA patients correlated with the Hammersmith Infant Neurological Examination (HINE-2) after 1.5 year of nusinersen treatment, and changes in the level of this miRNA were in line with improvements in motor function [199].

Also, three miRNAs (miR-181a-5p, -324-5p, and -451a), were significantly upregulated in serum from SMA patients among >100 miRNAs tested, which were differentially expressed in muscle samples from SMA patients compared to healthy controls [195]. These miRNAs contributed to a score predicting the phenotypic severity in SMA patients. The score was calculated based on the *SMN2* full-length (SMN2-FL) transcript levels, the *SMN2* gene copy number, and the age of the patient [195]. The combination of all these parameters enabled to markedly improve a predictive value of this score, though additional replicative and longitudinal studies are needed to confirm these findings.

Recent studies have revealed a significant number of new miRNAs that are of particular interest for exploring in the context of SMA [200,201]. Sixty-nine microRNAs were identified by NGS and were found to be dysregulated in plasma samples from patients with SMA types II and III compared to controls. Seven miRNAs (miR-107, miR-142-5p, miR-335-5p, miR-423-3p, miR-660-5p, miR-378a-3p, and miR-23a-3p) were associated with the response to nusinersen treatment at different time points. MiR-378a-3p levels were related to improved CHOP-INTED and HINE scores [200]. Sixty-six other miRNAs showed significantly altered expression in the CSF from six SMA patients treated with nusinersen compared to pre-treatment expression levels. Of these, fourteen miRNAs were predicted to regulate genes involved in the motor neuron survival and the pathogenesis of SMA [201].

Therefore, various classes of miRNAs that are essential for the development and function of nervous and muscular tissue have been found to be dysregulated in the serum, plasma, CSF, and muscles of individuals with SMA. These identified miRNAs reflect the underlying pathological mechanisms of the disease progression and could serve as presymptomatic biomarkers for the initiation of the disease. Additionally, the observed levels of expression of these miRNAs provide insight into the response to treatment, highlighting the potential utility of these biomarkers for predicting prognosis and monitoring therapeutic progress.

### 4.5. Neurofilaments

Neurofilaments (Nfs) are neuron-specific cytoskeletal components that maintain cell structural integrity [202]. Nfs are assembled from five subunits: neurofilament light chain (NfL), neurofilament medium chain (NfM), and neurofilament heavy chain (NfH) as well as alpha-internexin (INA) and peripherin (PRPH) in certain nerves [203]. Any of these subunits can be regarded as targets for measuring in a given neuropathological condition [202]. One of the key posttranslational modifications involved in Nfs formation and functioning is phosphorylation (pNfs) [204]. Mutations of genes encoding Nf proteins can induce neuronal pathology, axonal abnormalities, and neuromuscular disorders, such as Charcot–Marie–Tooth disease, and were shown to be associated with ALS, Parkinson’s disease, and Alzheimer’s disease [203,204,205]. Thus, Nf dysregulation may be a causative and modifying factor for certain neuropathological states [205,206]. At the same time, an aberrant Nfs level is often a secondary event in neuron pathologies, thus serving as a biomarker of nerve cells disruption or axonal loss. In neurodegeneration or a nerve injury condition, Nfs are released into CSF and blood and can be detected there by ultra-sensitive immunoassays [207].

A longitudinal study demonstrated that NfL levels were highly correlated between CSF, serum, and plasma, being significantly increased in samples from ALS patients compared to controls [208,209]. The blood NfL level was shown to remain stable over time and was determined as a strong predictor of ALS patients’ survival [208,209]. CSF pNfH and NfL levels were able to differentiate between ALS patients with slow and fast disease progression [210]. CSF and serum NfL and pNfH levels have also demonstrated valuable results as diagnostic, prognostic, and treatment response biomarkers for multiple sclerosis, Alzheimer’s disease, and other neurological conditions affecting the nervous system [202].

Taking into account such encouraging results, pNfH was explored as a promising pharmacodynamic/response biomarker in plasma samples from nusinersen-treated and sham-control infants with SMA as well as children without the disease [211]. Plasma pNfH levels were significantly increased in infants with SMA and inversely correlated with the age of symptom onset. The level of plasma pNfH subsequently decreased after treatment with nusinersen [211]. Serum (sNfL) and CSF (cNfL) neurofilament light chain also demonstrated an increased level in SMA children, especially those with two *SMN2* copies compared to those with more than two copies [212,213]. In all SMA children, sNfL levels showed a strong correlation with cNfL levels. The SNfL level was associated with motor function in patients with two *SMN2* gene copies [213]. The NfL levels measured in the CSF of patients with SMA type I carrying two *SMN2* copies decreased over time with nusinersen treatment, in accordance with improvement in motor function [212]. A study of serum pNfH and NfL levels in SMA patients aged 0–3 years with 2–4 copies of the *SMN2* gene revealed that NF levels were inversely correlated with the *SMN2* copy number and CMAP maximum amplitudes [23]. After nusinersen injection, a rapid decline in serum pNfH and NfL levels was observed in SMA patients with two *SMN2* copies and in four of the five patients with three *SMN2* copies [23]. Interestingly, only one of the seven patients receiving onasemnogene abeparvovec demonstrated downregulation of pNfH and NfL after treatment. The others showed an increase in Nf levels despite a progressive rise in CMAP values and achieved age-appropriate motor functions. Meanwhile, nusinersen provided to patients before gene therapy administration moderated the growth of Nf level [23]. These observations may provide additional insights into the mechanisms of the treatment effects, which should be thoroughly investigated in light of this biomarker.

The concentrations of CSF pNfH were inversely correlated with the CHOP INTEND score in SMA type I children after nusinersen treatment. Both NfL and pNfH levels were inversely correlated with HFMSE values in CSF samples from SMA type II and III patients [214]. In this study, a significant decline in NfL levels under nusinersen treatment was found only for patients with SMA type I. The decrease in pNfH levels was observed for both SMA type I and SMA type II pediatric patients [214]. In a recent retrospective study on the effect of nusinersen on pNfH levels in pediatric patients with SMA types I, II, and III who had 2–4 *SMN2* copies, the most significant reduction in CSF pNfH levels relative to baseline was observed between the initiation of treatment and the start of the maintenance therapy. This reduction was independent of the specific SMA type or number of *SMN2* gene copies [215]. Meanwhile, in this study, patients with two copies of the *SMN2* gene compared to those with three or four copies exhibited the highest levels of pNfH in CSF and serum at the pre-treatment stage as previously described [215].

At the same time, several studies examined pNfH and NfL levels in CSF from adolescent and adult SMA type II and III patients at the pre-treatment stage and after nusinersen injections and found no differences compared to control individuals at any of these stages [216,217,218,219]. This observation may be connected with the slow disease progression. Meanwhile, in the study of Faravelli and colleagues, the concentrations of pNfH and NfL in the CSF of SMA type III patients aged 9–74 years significantly decreased 6 months after nusinersen administration compared to baseline levels, which were similar to those observed in controls, although the decline in pNfH and NfL levels was minor and did not correlate with HFMSE values [220]. In another study, pNfH and NfL levels were measured in CSF and serum from adult patients with SMA types III and IV, and the effect of nusinersen treatment was examined [221]. Only the level of pNfH in CSF was found to decrease significantly under treatment, but this was not correlated with improvements in the clinical status of patients.

In summary, these findings indicate the high utility of measuring pNfH and NfL in serum or CSF as biomarkers for monitoring SMA onset, disease progression, and response to therapy, preferably in the youngest SMA patients who have no more than three *SMN2* copies.

### 4.6. Creatine Kinase and Creatinine System

Creatine kinase (CK) is a key enzyme in cellular energy metabolism that catalyzes the reversible conversion of creatine (Cr) and ATP to create the high-energy compound phosphocreatine (PCr) [222]. The CK is widely expressed throughout human tissues, with predominant enrichment in tissues with high energy requirements such as muscles. Up to 94% of total creatine (Cr + PCr) is detected in muscular tissue [223]. The muscular Cr and PCr are non-enzymatically converted to creatinine (Crn), which is the end product of creatine metabolism. Crn diffuses out of cells and can be easily detected in serum or urine [223,224].

Cr converts to Crn at a rather stable rate (about 2% of total Cr per day) and has been shown to reflect body muscle mass [223,225]. With loss of muscle mass due to muscular diseases, less creatine is converted to creatinine, leading to an increased ratio of creatine to creatinine [226]. Elevated CK activity and downgraded Crn levels, caused by muscle loss, were observed as being characteristic of various neurodegenerative and muscle diseases like ALS, DMD, and BMD as well as SBMA and SMA [223,227]. In ALS patients, higher CK levels have been correlated with longer duration of survival as well as slower progression rate of the disease [228,229,230,231,232]. Plasma and serum Crn concentrations have been shown to reflect the muscle waste and have been strongly associated with clinical progression and mortality risk in ALS patients, suggesting creatinine as a valuable biomarker for disease monitoring and prognosis [233,234,235,236]. Crn levels in DMD and BMD patients’ serum have been inversely correlated with disease severity, improving the prediction of functional performance [226,237,238]. Moreover, serum Crn has been found to be decreased in patients with SBMA before the onset of clinical symptoms, correlating with disease severity [239,240].

These observations in patients with primary or secondary muscle degeneration events disclose the potential benefits of Crn as a complementary biomarker for predicting disease progression and prognosis.

The study of CK activity in 504 SMA patients (138 type I, 127 type II, 144 type IIIa, and 95 type IIIb) revealed an association of higher CK levels with milder disease progression [241]. Thus, patients with SMA type III and especially IIIb possessed significantly higher CK levels compared to patients with SMA types I and II. Elevated CK activity in milder forms of SMA may be partially explained by the relatively greater muscle mass in patients with slower disease progression.

A study of the Crn level in 238 patients (49 type I, 97 type II, and 92 type III) revealed a significant association with the type of SMA, the number of *SMN2* copies, and HFMS values (comparing walkers, sitters, and non-sitters) as well as MUNE and maximum CMAP [242]. Overall, in this study and similar studies, higher levels of Crn were correlated with a greater number of *SMN2* copies and a lower level of SMA severity [242,243].

In a retrospective study, Crn serum levels were found to be strongly correlated with HFMS values during and after 3 years of nusinersen treatment in pediatric patients with SMA. Crn levels declined during the first 6 months of treatment but increased during maintenance treatment, being most pronounced in patients with SMA type II and III [215].

In adolescent and adult patients with SMA types II, III, and IV, Crn levels were also lower than in the healthy cohort and were correlated with HFMSE and RULM scores both at baseline and under therapy with nusinersen [218,244,245]. The Crn level was almost twice as high in SMA patients with four or more copies of the *SMN2* gene as in those with less than four *SMN2* copies [244,245]. In a study of Freigang and colleagues, CK level was found to be associated with SMA severity and motor function, being significantly higher in ambulatory patients [244]. CK level decreased during treatment, along with a gain in HFMSE scores. Moreover, this study demonstrated the predictive power of baseline Crn and CK levels, which were elevated in the group of treatment responders compared to non-responders, based on the HFMSE assessment [244].

A longitudinal study conducted by Zhao et al. found that Crn levels correlated with the Medical Research Council (MRC) scale, forced vital capacity (FVC), and CMAP [245]. Crn levels were shown to have the highest predictive power for HFMSE, MRC, and the 6 min walk test (6 MWT) compared to FVC, CMAP, body mass index (BMI), and *SMN2* gene copy number when used individually, and they enhance predictive value when used in combination [245].

Metabolome research revealed urinary Crn among a wide spectrum of metabolites as the most significant one, distinguishing SMA patients from control individuals [246]. At the same time, no changes in the level of this molecule were detected under nusinersen therapy [246].

Overall, CK and especially Crn are suggested as promising predictive and monitoring biomarkers for SMA. The results of CK-level measurements were more controversial, while the Crn level appears to have been better studied in recent years, with some encouraging results observed. Among the advantages of using Crn as a serum biomarker are its simplicity, low cost, and non-invasiveness. These characteristics allow for repeated testing and longitudinal follow-up, making Crn level a convenient option for monitoring [247].

## 5. Conclusions

In the treatment era, it is of great importance to optimize therapies for patients with spinal muscular atrophy (SMA). This involves identifying biomarkers that can predict disease progression and response to treatment as well as monitoring changes in patient outcomes during therapy. Furthermore, new therapeutic approaches for SMA are being developed, and biomarkers that can be used to assess these treatments preclinically are particularly valuable.

Certain investigated biomarkers, whose values were found to correlate with SMA severity and clinical course, have a potential to become predictive or prognostic. Such biomarkers may be linked to primary (motor neuron) or secondary (muscle) pathogenic alterations. Some of them can be easily detected in patients’ blood. Information derived from the measurements of various biomarkers can be consistently applied to consecutive time periods in managing patients. Therefore, the genetic background, which includes information about the type of *SMN1* mutation (deletion, conversion, or point mutation) combined with the number of *SMN2* gene copies, and the analysis of positive and negative point mutations within these genes plays a diagnostic and prognostic role. In addition, some newly discovered biomarkers can answer the question of whether disease progression has already begun even if clinical outcomes appear normal. Furthermore, biomarkers for treatment response and monitoring of disease development are used.

In this review, we provided information on the most promising biomarkers and their sensitivity, limitations, and possible applications (Table 1). Also, multiple data on novel putative biomarkers for SMA and therapeutic responses were derived from omics analysis [248]. Further validation studies are needed to confirm the relevance of these findings.

In summary, in order to obtain the most comprehensive characterization of the clinical state of patients with SMA, the progression of the disease, and the dynamic response to treatment, a combination of various biomarkers appears to be the most promising approach.

## Figures and Tables

**Table 1:** List of SMA biomarkers, their functions, and correlations with different parameters.

Biomarker	Findings on Biomarkers in SMA Patients	Functions as an SMA Biomarker
Electrophysiological biomarkers		
CMAP and MUNE	- correlate with age, SMA type, *SMN2* gene copy number, and motor function	- prognosis of the functional outcome
		- monitoring the disease progression and the effect of therapy (=monitoring)
		- prediction of the clinical improvement after therapy
MUNIX	- correlates with muscle strength and SMA severity	- monitoring
MUSIX	- is increased in SMA patients compared to healthy controls	- monitoring
Functional biomarkers		
Spirometric measures	- differ between SMA types	- prognosis for autonomous breathing
Dynamometric measures	- correlate with motor function	- monitoring
Imaging biomarkers		
Ultrasound measures	- correlate with SMA type and motor function	- monitoring
MRI measures	- correlate with SMA type, duration of the disease, and motor function	- monitoring
		- prediction of the clinical improvement after therapy
EIM measures	- correlate with SMA type and motor function	- monitoring
Molecular biomarkers		
*SMN2* gene copy number	- correlates with SMA type, motor function, and duration of survival	- prognosis of the disease progression - prediction of the clinical improvement after therapy
*SMN2* gene mutations and polymorphisms	- impact SMA severity	- prognosis of the disease progression
*SMN* transcripts level	- differs between SMA types	- monitoring - preclinical studies
SMN protein	- is associated with SMA type and the severity of denervation	- monitoring
Gems number	- correlates with SMA type	- monitoring - preclinical studies
microRNAs	- differ between SMA and healthy - correlate with motor function	- disclosure of new pathological pathways - prediction of the clinical improvement after therapy - prognosis of the disease progression - monitoring
Neurofilaments	- correlate with the SMN2 copy number and motor function	- monitoring
Creatine kinase	- is associated with SMA severity and motor function	- monitoring - prediction of the clinical improvement after therapy
Creatinine	- correlates with the SMN2 copy number, SMA type, and motor function	- monitoring - prediction of the clinical improvement after therapy
